# A Qualitative Study Exploring Factors Associated with Retention in HIV Care among Women with HIV in a Large HIV Clinic in Lagos, Nigeria, after Implementing the Test and Treat Policy

**DOI:** 10.1155/2022/9074844

**Published:** 2022-08-09

**Authors:** Omoladun O. Odediran, Oluwakemi O. Odukoya, Mobolanle R. Balogun, Jonathan A. Colasanti, Alani S. Akanmu

**Affiliations:** ^1^Department of Community Health and Primary Care, College of Medicine, University of Lagos, Lagos, Nigeria; ^2^Department of Community Health and Primary Care, College of Medicine, University of Lagos & Lagos University Teaching Hospital, Idi-Araba, Lagos, Nigeria; ^3^Emory University Division of Infectious Disease, Atlanta, GA, USA; ^4^Department of Haematology and Blood Transfusion, College of Medicine of the University of Lagos, Lagos, Nigeria

## Abstract

**Background:**

In Nigeria, various sociocultural and economic factors may prevent women from being retained in HIV care. This study explores the factors associated with retention in care among women with HIV in a large HIV clinic in Lagos, Nigeria, under the Test and Treat policy.

**Methods:**

Women living with HIV/AIDS (*n* = 24) enrolled in an HIV study at the AIDS Prevention Initiative in Nigeria (APIN) clinic in Lagos, Nigeria, were interviewed from April 1 to October 31, 2021, using a semistructured interview guide. Interviews were audio-taped, transcribed verbatim, and the themes were analyzed using the framework of Andersen and Newman's Behavioural Model for Healthcare Utilization.

**Results:**

The mean age of the respondents was 37.4 ± 9.27 years. The identified themes were as follows: being aware of the antiretroviral medications and their benefits, the household's awareness of the respondents' HIV status, and the presence of social support. Other themes were the presence of a dependable source of income and the ability to overcome the challenges encountered in obtaining income, ease of travel to and from the clinic (length of travel time and transportation costs), securing support from the clinic, challenges encountered in the process of accessing care at the clinic, and the ability to overcome these challenges. Also mentioned were self-perception of being HIV positive, motivation to remain in care, linkage to care, and intention to stay in care.

**Conclusion:**

Several deterring factors to retention in HIV care, such as nondisclosure of status, absence of social support, and clinic barriers, persist under the Test and Treat policy. Therefore, to achieve the “treatment as prevention” for HIV/AIDS, especially in sub-Saharan Africa, it is essential to employ strategies that address these barriers and leverage the facilitators for better health outcomes among women with HIV/AIDS.

## 1. Introduction

Nigeria has the third-largest HIV epidemic globally and one of the highest incidence rates in sub-Saharan Africa [1]. The prevalence of HIV in Nigeria is 1.3% among the adult population, representing 1.6 million Nigerian adults living with HIV at the end of 2020 :  960 thousand of these cases are women, while 650 thousand are men [2]. The Test and Treat policy was introduced into the guidelines for managing HIV/AIDS by the Federal Ministry of Health in December 2016. This policy was implemented in response to the Treat All initiative of the World Health Organization (WHO) [3]. The policy provides universal access to comprehensive HIV treatment and care and reiterates that all people with HIV are eligible for antiretroviral therapy (ART) immediately after diagnosis.

Retention in HIV medical care measures one's engagement with care [4]. Multiple methods for estimating retention in care are often based on the number of HIV clinic visits attended regularly [4]. The ability to be retained in care for HIV-positive individuals is critical to achieving good treatment and health outcomes and curbing the incidence of HIV infection [4]. Recent studies have shown that lower retention in HIV care is a significant barrier to optimizing HIV care, leading to a worse health outcomes and increased HIV infection [5]. Retention in care and adherence to treatment is critical for favorable outcomes in HIV care, making it imperative to assess the retention in care under this new policy and the factors associated with patients' retention in HIV care, especially in settings such as Nigeria, where the loss to follow-up can be common [6]. Program studies in Nigeria often report more than a 20% loss to follow-up greater than 12 months before the Test and Treat policy was implemented [7, 8].

According to the United States Agency for International Development [9], potential barriers to retention in care include side effects of the ARTs, perceived stigma of antiretroviral drugs, and difficulties accessing HIV care centers during operating hours of work. Others include poor service quality, the discriminatory attitude of the clinic staff, depression, anxiety, and lack of family support. For persons with HIV, being retained in care is vital for achieving good health outcomes and preventing HIV transmission. Women make up the more significant proportion of people with HIV/AIDS and need to be supported in every way possible to ensure that they remain in care and have positive health outcomes [10, 11].

Nigeria has several public health challenges due to the lack of appropriate health policies, poor resource allocation, and limited resources. HIV/AIDS remains a significant public health issue in Nigeria, with women disproportionately more affected, accounting for 56.5% of infected cases [12]. Gender inequality, often perpetuated by cultural and societal norms, remains integral to HIV/AIDS transmission [12]. Young women are more vulnerable to the disease, while older women and young girls are disproportionately affected by the burden of care for other family members with HIV in the wake of the scourge [12]. Nigeria has one of the highest unequal balances of power between men and women globally. Gender power imbalances mean that women often face barriers in dictating their sexual partner selection, use of contraception, number and spacing of children, and their healthcare, all of which put them at greater risk of HIV [13].

This study aimed to explore the facilitators and barriers to retention in care among HIV-positive women after the Test and Treat policy was implemented in Nigeria. Using a qualitative approach, we interviewed a sample of women seeking HIV care and enrolled as patients at the AIDS Prevention Initiative in Nigeria (APIN) treatment center, Lagos University Teaching Hospital (LUTH).

## 2. Methods

We conducted an exploratory qualitative study to examine the facilitators and barriers to retention in HIV care among women seeking HIV care at the AIDS Prevention Initiative in Nigeria (APIN) clinic in Lagos, Nigeria. This clinic is located at the Lagos University Teaching Hospital (LUTH). It is one of the largest HIV clinics funded by the United States Presidential Emergency Plan for AIDS Relief (PEPFAR) in Nigeria.

From April 1, 2021, to October 31, 2021, we scheduled and conducted interviews by individual phone calls with women retained in HIV care using a pretested interview guide with open-ended and probing questions adapted from the literature review [14].

### 2.1. Participant Recruitment

To be eligible for the study, respondents must be female adults clinically diagnosed with HIV and must have been enrolled in HIV care at the APIN clinic between January 1, 2017, and December 31, 2019. The women must have been in care for at least 12 months when the interviews were conducted. According to APIN treatment center guidelines, retention in care was considered as women known to be alive and attending a minimum of two (2) HIV care visits greater than 90 days apart within 12 months after ART initiation [15]. Recruitment occurred through a purposive sampling approach. We obtained a list of all patients registered at the clinic between the stipulated periods to identify respondents. This list was filtered to obtain the compilation of HIV-positive women retained in care at the clinic for twelve months and over. The final sample of eligible women was 252. Fifty of the eligible women were randomly selected and were contacted by phone, informed of the study and its objectives, and asked about their interest in participating in the study. For the women who showed interest, a study team member contacted them by phone calls to inform the women about the study method, its scope, and objectives. The team member called back after one week to obtain consent from the women, and interviews were scheduled at a convenient time for each woman. Interviews were continued until no new information was obtained from the women.

### 2.2. Data Collection Tools and Techniques

The interview guide had five sections: Section one contained questions on demographics. Section two explored the knowledge of the women on ARTs and the benefits and frequency of taking the medicines. Section three explored the presence of social support and how the social support assists the women to remain in care, the source of income, and challenges that may arise in earning income for daily living, and motivation for staying in HIV care. Section four was in two parts: the first part dealt with the ease of transportation to the clinic, cost of transportation, and travel time, while the second part of the section attempted to explore the perception of the women on the quality of care received at the treatment center and how they feel the services can be improved to ensure continued retention in HIV care. Other questions explored the challenges they may experience in accessing care and how they overcome some of these challenges. Section five contains questions on the intention to remain in HIV care.

Using Andersen and Newman's Behavioral Model for healthcare utilization (ANBM) [16–18], we defined and analyzed the factors that may influence retention in HIV care. This model provides a framework for understanding how patient and environmental factors may impact retention in HIV care and outcomes. ANBM is based on three major components, which are as follows:

#### 2.2.1. Predisposing Factors

Individual predisposing factors include the demographic characteristics of age and sex, social factors such as education, occupation, ethnicity, social relationships, values, and knowledge related to health and health services.

#### 2.2.2. Enabling Factors

Financial capability and institutional factors enable services utilization. Individual financial status involves the disposable income and wealth at an individual's disposal to pay for health. Institutional factors deal with availability and access to a regular source of care.

#### 2.2.3. Need Factors

At the individual level, these include perceived need for health services (i.e., how people view and experience their general health, functional state, and illness symptoms) and evaluated need (i.e., professional assessments and objective measurements of patients' health status and need for medical care).

A total of twenty-four (24) interviews were conducted between April 1 and October 31, 2021, and each lasted 35 minutes on average. The interviewer was trained to prevent interference of personal bias, judgments, or assumptions. All interviews were conducted in English; field notes were made during the interviews and used to augment analysis. Incentives were given to the participants in the form of airtime phone credits. Interviews were confidentially conducted with the informed consent of the participants. We obtained informed consent from the women to audiotape their responses by phone.

### 2.3. Data Analysis

Responses of the participants were audiotaped and the information later transcribed verbatim. The interviewer and one trained research assistant reviewed the transcription for accuracy. The data were analyzed manually using narrative and content analysis by two research assistants who participated in a two-day training on developing a qualitative codebook and identifying themes using our study objectives. We applied the generated codes to the transcribed responses of the participants, separating the responses to each of the questions using Microsoft Excel; recurring themes were identified and presented cohesively in line with the study objectives and analyzed using the ANBM of healthcare utilization. Participant quotations were presented in line with the themes, with each quote deidentified. Central themes are presented in Table1.

Ethical approval was obtained from the Health and Research Ethics committee (HREC) of the College of Medicine, University of Lagos, with the approval number CMUL/HREC/01/21/791.

## 3. Results

### 3.1. Participant Demographics

A total of 734 women were registered in care; 411 were still in care at the end of 2019, of which 252 were eligible for the interviews. Fifty of the women were randomly selected and contacted for the interview. Twenty-four (24) in-depth interviews were conducted among HIV-positive women retained in HIV care. The ages of the women ranged from 18 to 56 years, with a mean value of 37.4 ± 9.27 years. Most women had postsecondary education (66.7%), 70.8% were employed, and 41.8% were married.  Table 2 provides the demographic characteristics of the respondents.

### 3.2. Themes

Using the ANBM (Table 1), we summarized the themes and the subthemes of the determinants of retention in care as perceived by the respondents.

#### 3.2.1. Predisposing Factors

Several key themes emerged from the qualitative data that describe the predisposing factors women have toward knowledge of and benefits from taking ARTs.

### 3.3. Knowledge and Attitude of the Women to ART

#### 3.3.1. Most Women Are Aware of ARTs and Their Benefits

Sixty-eight percent (68%) of the respondents knew what ARTs are. Some could not describe the function of the drugs in clear terms, but they were able to state that they no longer fall ill if they are on ART. Some participants reiterated this in the following excerpts:

“I know that if we stop taking the drugs, we will start falling sick again.” (KI2)

“…what I understand when I started taking my drugs, not that the drug will make it (HIV) to go, but it will make somebody live for the meantime, not forever.” (KI4)

When asked about their perception of the benefits of taking the drug for women with HIV and the people around them, all the respondents mentioned that taking the ART benefits people living with HIV/AIDS (PLWHA). Some of the mentioned benefits include better health, more strength, and the ability to carry out activities for daily living as stated below:

“For me, I see myself as a living person when I am taking the drug because all those symptoms that are there before are going gradually.” (KI3)

“…so that the virus does not get out of hands to be full-blown AIDS.” (KI6)

In comparison, 28% of the women were not aware that taking ART regularly has its benefits for the people around them. Some responded:

“No, they do not know I am positive.” (KI1)

Others mentioned that it is beneficial to the people around them:

“You are not spreading the virus to them, so you are keeping them safe.” (KI6)

Ten percent (10%) of the respondents mentioned the incidence of side effects as one of the disadvantages of taking ARTs. When asked how they could overcome this challenge, the women said they notify the clinic about the challenges with the drugs. The drugs are either changed, or the women are taught some coping mechanisms with the side effects once the clinic is notified:

“The one I took before if I take it and I do not eat on time, it will weaken my body, and I stool. I complained, and they changed the drug for me.” (KI8)

When asked how often they feel the ARTs should be taken, 90% of the respondents said every day, but some of the women admitted skipping a few doses due to lack of access to where the drugs were kept or tiredness.

“Every day, but I will not lie; I do not take it every day, sometimes I am far away from where the drug is. I have been away from it for up to 2 months.” (KI10)

“Every day, I take at night, and I do not forget, no matter how tired I am, there is timing for it. Occasionally I miss it, maybe once or twice a month when I am tired and sleep off. I will not lie to you.” (KI11)

### 3.4. Social Norms and Perceived Control

#### 3.4.1. Household Awareness about Respondents' HIV Status

The women were asked if anyone in their household knew about their HIV status; 79% of respondents affirmed that their family members were aware of their status. The respondents gave reasons such as informing certain household members based on trust. Some household members knew because they were present during the screening and diagnosis process or due to their involvement in taking care of the sick.

“My older brother and my older sister, I told my sister two weeks after my diagnosis. I have to tell her, and I trust her. She has been very supportive; even when I cut my hair, she cut her hair.” (KI14)

“My mum and my sister, it was a long story. …it was my mum that took me to the hospital, so she knew automatically.” (KI6)

However, some respondents (21%) stated that no one in their household knew about their status because they do not trust anyone with such information to avoid being stigmatized or to prevent divorce from their spouses.

“I do not want, people I have are lousy, so I do not want to tell anyone.” (KI22)

“Nobody knows about my status; my husband does not know; if he knows, it means I will be sent away from his house.” (KI6)

“It depends on the kind of home you come from. If misunderstanding comes up now, they can use it to abuse you, so let me hide my head. So I did not tell anybody.” (KI4)

#### 3.4.2. Presence of Social Support

Thirty-seven percent (37%) of the women responded that they do not have anyone they depend on for social support. The respondents cited the inability to trust other people with information about their HIV status as the primary reason they do not have social support.

“I cannot tell my friends o! My family members disowned me talk less of my friends. (Probe-but, your mum, is a family member?)Yes, she is different, and even at that when I offend her, she uses it to insult me. I cannot tell anyone again, except maybe I met someone at the treatment clinic, and we became friends. I do hide my drugs; it is my secret.” (KI19)

*(1) Enabling Factors*. The key themes were as follows: the ability to pay, availability and accessibility of the treatment facility, and treatment facility factors.

### 3.5. Ability to Pay

#### 3.5.1. Sources of Income and Challenges Encountered in Obtaining Income for Daily Living

Many respondents (62.5%) stated that they depend on their spouse/partner or other family members for income,

“Yes, I am helping my cousin out at least, and I am dating someone. He supports me with transport money.” (KI1)

“Yes, my husband (supports me), because we do everything together” (KI3)

Thirty-seven percent (37%) of the women responded that they have an adequate source of income and do not depend on other people.

“No, I do not depend on anybody. I sustain myself with my business.” (KI5)

On the challenges that HIV-positive women may face with obtaining income for daily living, 66.7% of the women responded that they do not have any challenge in getting income for everyday living, while 33.3% gave reasons such as stigmatization, loss of job, other family responsibilities, and poor economic downturn.

“Where I worked before, because of my frequent illness, some fraudulent guys signed documents I did not sign and obtained company money, and I was laid off. I got a new job; coming to the clinic is challenging, especially when trying to hide your status.” (KI17)

“I had a job before I fell sick; I did not resign; I took an extended leave to care for my health. I tried to sue the initial hospital I visited; that was how my employers discovered my status. When I apply for a job, they say no chance once they know my status” (KI5)

“For now, I am taking care of my mum, and I eat whatever she eats. I pray she gets ok so that I can try to start up my life again. I have been with her since December 2019 now.” (KI12)

“My shop just got demolished, so I am looking for a new place to trade.” (KI11)

### 3.6. Availability and Accessibility of Facility

#### 3.6.1. Difficulties/Challenges Encountered in the Process of Accessing Care at the Clinic

Sixty percent (60%) of the women stated challenges in access to care at the clinic. These challenges include transportation costs, delays at the clinic, taking time off work to be at the clinic, cost of laboratory tests, secrecy, and other family responsibilities:

“Like me, for more than five months I was not able to come because of no transport money, talk less of money to run my test.” (KI1)

“Transportation cost and taking excuses from work; as I said earlier, it already cost me one job.” (KI7)

“I said I work in a company; when I come to the clinic, they do not attend to us on time, you will be there 2 o'clock, 3 o'clock.” (KI8)

“Just the regular Nigerian system; (probe: Please clarify?) -like you going early and they change your file for their friends'.” (KI9)

“Some people, if they are secretive to their partner or somebody next to them, it will be tough for you to go to the clinic and get your drugs, except you work and you do not work with your husband, or else how will you come to the clinic secretly.” (KI7)

Forty-five percent (45%) of the respondents mentioned that they plan and organize transportation to the clinic before the set date or save up until they can afford the transport and laboratory test costs, often leading to missed appointments; others ask for a rescheduled clinic appointment or take unpaid leave for the day to attend the clinic.

“So what I do is to get a vehicle that will agree before my clinic day so that I do not miss my appointment.” (KI6)

“I save up the transport fare, and I try to be as discrete as possible at work.” (KI8)

“I come when I can, but before my drugs finish.” (KI16)

“It has not been easy, especially when appointment clashes with work. Once I know the next appointment will clash, I ask the doctors to reschedule it for me- during midterm breaks and public holidays. Or I give excuses at work, highest they will deduct the day's pay from my salary.” (KI3)

#### 3.6.2. Ease of Access to the Clinic, Travel Time, and Transportation Costs

Some of the women (66.7%) responded that getting to the clinic is challenging; they spend over an hour on average as travelling time to the clinic and between an average of 1,000 and 1,500 naira (approximately 2-3 USD) on transportation costs to and from the clinic.

“It is not easy; sometimes I trek to reduce transport cost.” (KI2)

“Transportation is not easy; I live at Ajah, so sometimes I do not come for the appointment.” (KI4)

“When I lived in Lagos from Ketu, I think I spent maybe 1000 naira plus, at times my husband would carry me before he got to know about my status. When I moved to my sister's place from Shagam, I sometimes spent 3000 naira on transport to and fro; if I stay late and start pricing, maybe about 2000 naira. It takes about 2 hours to get to the hospital.” (KI7)

### 3.7. Healthcare Facility Factors

#### 3.7.1. Presence or Absence of Support from the Treatment Clinic

The women were asked about their perception of the support received from the clinic; most of the respondents (66.7%) said that they had received adequate support from the clinic. The support mentioned includes ART counselling, financial aid, job counselling, and emotional support.

“They have been supportive; there was a time they gave some of us transport fare back. The doctor (mentioned) gathers the youths, advises them, and does get-togethers for them. The youths need such, and she is trying with her money. I commend them.” (KI12)

“They give me advice, and I will never forget that. I go to see the counsellor. Even in the pharmacy, they give advice. Even if I do not have money, they still help out. I have been going to meetings for adolescents. E.g., operation triple zero. We do get-together parties, and we share our stories and go for outreaches.” (KI7)

Some women (33.3%) responded that they do not receive adequate support from the clinic, citing reasons such as unfriendly and insulting staff, preferential treatment, and delays.

“They (the clinic staff) are very insulting; they even made me tired of coming to that place. If I remember my appointment, I will be sad. They are discriminating against somebody (they discriminate against somebody). That is why I said if there is a place they are selling the drugs; I will buy for myself and not bother to come.” (KI4)

“They tell you no network to check your viral load, no doctor, sometimes you spend the whole day, sometimes you will be there till…, except I tip them that is when I can leave on time. I wish there were an injection; you hear patients complaining about the drugs where you sit.” (KI3)

“Not so bad, but they should be a bit more accommodating and faster. They waste a lot of time. Some of them are always kind of edgy and act like they are always angry, but the doctors are nice.” (KI6)

When asked about any other kind of support they would like to receive at the clinic, the respondents mentioned reducing waiting time, more privacy, and the reduction of the cost of laboratory tests.

“Timing, some people work, and you come around 7.30 am to 8 am and leave at noon. I feel they waste time. The accountant is not there even when you want to pay for the test. Since it is an appointment, you should not be kept for more than 2 hours. For people working when you take excuse from work every six months, and you do not return for the day, you are playing with query.” (KI5)

“If they can make it private, it is too crowded. The number they see is too much. When they shout your name, everyone sees you before you walk the whole length. I once saw my neighbor from the market and had to dodge.” (KI19)

“Like the test we do, and we pay 5000.00 naira (approximately 10USD), it is expensive before we do not pay, they should do it for free or for a token, the 5,000.00 naira is too high.” (KI6)

*3.8. Need Factors*. The significant themes were the individual's perception of health, perceived needs, and evaluated needs.

### 3.8. Individual's Perception of Health

#### 3.8.1. Individual's perception of health/Self-Perception of Being HIV-Positive

On exploring the feeling of the respondents about being diagnosed with HIV and how they handled the news, the women expressed a range of emotions from being sad, depressed, angry, surprised, or shocked. Some respondents (24%) found out about their HIV status from mass screening exercises (20%), while some were from opportunistic testing during child immunization visits. In comparison, others (56%) fell ill after contracting the virus.

“I was sad; I wanted to bring the hospital down. My husband was sick, and he did not come clean. The doctor suspected it was HIV and asked me to test. It was positive. I lost my husband in 2017. He never disclosed that he had HIV.” (KI16)

“I was not happy o, because it was something I was not expecting; I went to church for medical screening- malaria, typhoid, and HIV. When it came out, they did not give it to me. When my sister saw it, she did not believe it; she asked me to go to another hospital for a test. That was how I came to LUTH.” (KI4)

“I was depressed. It was during my child immunization, and we already had two kids. I was told that my baby is positive.” (KI13)

All the respondents expressed that they currently feel better and are hopeful for a cure for HIV/AIDS.

“I am better; if I tell you I have it, you will not believe; I am healthy. I take my drugs, and I eat well.” (KI6)

“You always still have the stigma. I feel if it has gone, I will be much better.” (KI7)

“There are plenty diseases now- cancer, diabetes, etc. I use that to console myself that the HIV is not a death sentence. I just take it as normal; people who do not have HIV also die.” (KI12)

### 3.9. Perceived Needs

When asked about their motivation for remaining in care, the reasons given by the respondents were as follows:

#### 3.9.1. Personal Drive and Focus on Health and Life Goals (25.7%)

“So the motivation is to be healthy so that you can be at the same level as other people or even better. “I want to live, and I want to fulfill my purpose in life. I have to.” (KI9)

“So long as you are on the drug, it does not stop you from getting married and having children; we see examples at the clinic.” (KI3)

#### 3.9.2. Trust in the Healthcare Personnel (20%)

“A female doctor moved me to check so that I do not fall into what my husband suffered. She keeps check on me from time to time. I also want to be there for my children and grandchildren.” (KI14)

#### 3.9.3. To be Able to Care for Their Children (66.7%)

“…for my daughter's sake. She has no father.” (KI3)

“There are a lot; one will be okay, they said if you do not take it the disease will come back, one will live long and will be able to take care of my children.” (KI1)

“I want o to be ok; I do not want anything to happen to me because my children are still small.” (KI5)

### 3.10. Evaluated Needs

#### 3.10.1. Linkage to Care

On time from diagnosis to linkage to care, 25% of the respondents mentioned that they were started on ART the same day. In contrast, the remaining 75% said a time lag of about one or two weeks for the results of laboratory investigations to be ready before they were placed on ART.

“I started immediately. I was informed about my status. I was at LUTH because my baby was sick...” (KI16)

“The following week, I was pregnant, and I started immediately. My viral load dropped drastically.” (KI2)

#### 3.10.2. Intention to Remain in Care

The women responded that the first step taken after a clinic appointment is to check for the date of their next clinic appointment and then keep the drugs in the usual place to facilitate easy access and out of prying eyes.

“I check the next date for my appointment, and I go home. I keep the drug where I used to keep it from prying eyes.” (KI5)

“I remove it from the pack and check for my next appointment, and off I go. I have a place I keep them at home.” (KI9)

## 4. Discussion

Several factors interplay to influence women's retention in HIV care in Nigeria. Women's existing knowledge and beliefs about HIV and ART facilitated retention in their HIV care. The respondents in our study mentioned some of the benefits of taking ART to include improving health and having the strength required for daily living. A similar qualitative study in an HIV clinic in Southern Ethiopia identified personal factors such as misconceptions about HIV and ART as deterring factors to retention in care. Participants in their study reported that persons who received a positive HIV result were often unaware that ART and other medical interventions could help them remain healthy [19].

Most of the respondents who had disclosed their HIV status to their partners or other household members mentioned that they had adequate support to remain in care. In contrast, HIV status disclosure has been a devastating experience for a few, leading to stigmatization. Several studies have demonstrated that disclosing an individual's HIV status to household members enhances retention in care and provides social support [20, 21]. Although disclosure of HIV status may have dual effects regarding accessing social support, the benefits of disclosure overwhelmingly outweigh potential adverse effects [22, 23]. A cross-sectional, descriptive study conducted in Kwa-Zulu Natal, South Africa, in 2008 explored the relationship between social support, HIV-related quality of life, and adherence and concluded that having close friends and family was significantly associated with a greater sense of social support [24]. Our study findings also suggest that a supportive social network is essential for retention in care among women living with HIV/AIDS. However, a prospective observational cohort study of persons newly diagnosed with HIV infection carried out among 168 HIV-positive individuals followed up for over one year in Texas noted that social support may not be sufficient to ensure success across the HIV care continuum [25].

Enabling factors mentioned by the participants include the following: the ability to pay (sources of income and the challenges encountered in obtaining income for daily living), availability and accessibility of care facility (such as ease of travel to and from the clinic, travel time, and transport costs), and health facility factors (presence or absence of support from the clinic, and challenges in accessing care and the ability to overcome these challenges). We observed that the ability to be financially self-sufficient or have a dependable income source plays a significant role in retention in care for the respondents. Poverty and economic insecurity are known barriers to routine access to HIV care and treatment services. With the global HIV burden higher in resource-limited settings, direct and indirect costs of seeking care can prevent retention in HIV care [26].

High transportation costs, time away from income-generating economic activities, and the costs of medical services create barriers to care and treatment, which is more challenging for those who are not economically stable [26]. In as much as the direct costs of ART are free, there is still the ancillary care cost, such as the cost of laboratory tests, which is 5000.00 naira (about 10 USD). Some participants consider the laboratory test costs as a deterring factor to retention in HIV care at the clinic. Similarly, a study conducted in Botswana among HIV-positive individuals and their health care providers noted that if cost is removed as a barrier, adherence will be predicted to increase from 54% to 74% [27]. In Nigeria, where 40% of the citizens live below the poverty line [28], food insecurity may affect adherence in multiple ways. There will be trade-offs will be between paying for food and accessing treatment, leading to missed appointments [29].

The respondents in our study mentioned clinic barriers such as long wait time, poor attitude of some clinic staff, and lack of privacy. Other barriers mentioned were nondisclosure of HIV status, time away from work, transport costs, and laboratory test costs. Lifson and colleagues [19] noted that negative experiences of receiving HIV care, which may adversely affect retention in HIV care, can be provider- and system-based. Negative provider interactions included the perception that doctors or nurses are impatient or do not express a welcoming attitude. Also included are concerns about the lack of confidentiality by clinic staff, including counselors. Other adverse health system factors included the frequent change of clinicians, which may result in changed treatment plans; a poor medical records system; overcrowding and shortage of chairs in waiting rooms; and long waiting time for appointments, test results, or other services. For example, because results for laboratory tests such as CD4 count were not immediately available, patients might have to return several days to a week after an initial visit for counselling about their results [19].

Need factors were an individual's perception of health (self-perception about being HIV-positive), perceived need to remain in HIV care (motivation for staying in HIV care), and evaluated needs (linkage to care and intention to remain in care). On self-perception of being HIV positive, the respondents expressed various emotions when told about their HIV diagnosis, but finally accepting their HIV status was seen as a positive influence on remaining in care. Similarly, a study conducted in the Shiselweni region of Swaziland exploring the influence of acceptance and denial on linkage to HIV care concluded that acceptance of HIV-positive status appears to be crucial to the success of the Test and Treat initiative. At the same time, disbelief on receiving a positive diagnosis can prevent accessing HIV care [30].

The respondents in our study stated that they take their medications regularly except for occasional missed doses. The motivation for attending the clinic and taking the ART was to be healthy and live long, to be able to care for their children, and also as a result of encouragement from the healthcare workers at the clinic. A qualitative study conducted in three Ryan White-funded HIV clinics in Philadelphia, USA, in 2015, mentioned the perceived need for retention in HIV to include the belief by the respondents that the medications are keeping them alive [31].

On the time of clinical diagnosis to linkage to care, the respondents mentioned that they were linked to HIV within two weeks of diagnosis. This is an essential step to facilitate retention in care under the test and treat policy. It has been demonstrated in several studies [32–34] that immediate linkage to care and commencement of ART is essential to achieving and maintaining viral suppression. Retention in care and adherence to ART have been independently associated with good long-term HIV outcomes [35]. Still, the underlying behavioral factor is the intention to remain in care. Our respondents mentioned looking out for their next clinic appointment once they receive their medications and keeping the newly collected ART packs in the usual place not to forget to take them. A study carried out among 244 adults in two HIV clinics in Houston, Texas, noted that only intention to adhere to HIV treatment remained a statistically significant predictor of antiretroviral adherence after adjusting for other confounders [36].

A study assessing the status of HIV-positive individuals considered lost to follow-up at APIN clinic before the implementation of the Test and Treat policy (from 2010 to 2014) noted that the primary reported reasons for HIV-positive individuals' discontinuation from the clinic were long distance and high transportation costs to the clinic, unfriendly staff, and feeling healthy [37].

### 4.1. Study Limitation

There are several limitations to this study. The HIV-positive women retained in care were purposively sampled, which can be prone to selection bias and may reduce the generalizability of the results. Additionally, participants' responses may have been influenced by social desirability bias. However, the interviewer was trained to ensure confidentiality and avoid judgmental reactions to minimize this risk. Our interviews were limited to women retained in care, so while we could garner some ideas of potential barriers to care, the absence of poorly retained patients decreases our ability to draw strong conclusions about true barriers. Finally, as with all studies of a purely qualitative nature, the findings of this study may not be generalizable to other populations, particularly as the respondents are all women, and clinical practices and geographic and cultural environments may vary by gender.

## 5. Conclusion

Test and Treat hold great promise to deliver ART to more persons with HIV. However, many of the same barriers to long-term retention are present in the context of where the patients live and attend clinic. Our results emphasize the need to educate patients about HIV and treatments; understand patients' social environment, disclosure, and levels of stigma; understand patients financial and transportation situations; and improve customer service and efficiency at the level of the clinic. There is the need to seek contextually appropriate interventions to address these factors to reduce the transmission of HIV infection and ensure good health outcomes for HIV-positive women.

## Figures and Tables

**Table 1:** Summary of themes and sub-themes using Andersen and Newman's behavioral model of healthcare utilization.

Predisposing factors	Enabling factors	Need factors
Knowledge and attitude of the women to ART: *Knowledge about the function of ARTs and the perceived benefits of taking them as directed by the clinic*	Ability to pay: *Sources of income and challenges encountered in obtaining income for daily living*	Individual's perception of health: *Self-perception of being HIV-positive*
Social norms and perceived control: *Household awareness about respondents' HIV status* *Presence of social support*	Availability and accessibility of facility: *Ease of travel to and from the clinic: length of travel time and transportation costs*	Perceived needs: *Motivation for remaining in care*
	Healthcare facility factors: *Presence or absence of support from the treatment clinic* *Challenges encountered in the process of accessing care at the clinic* *Ability to overcome challenges in accessing care at the clinic*	Evaluated needs: *this should be linkage to care intention to remain in care*

**Table 2:** Participant characteristics.

Variable	Frequency (*n* = 24)	Percentage
Age group (years)		
≤30	4	16.7
**31**–**40**	**13**	**54.2**
41–50	5	20.8
>50	2	8.3
Mean ± SD	37.40 ± 9.27	
Marital status		
Single	7	29.1
Married	**10**	**41.7**
Separated	4	16.7
Divorced	1	4.2
Widowed	2	8.3
Highest level of education		
Primary	2	8.3
Secondary	6	25
Post-secondary	**16**	**66.7**
Employment status		
Employed	**17**	**70.8**
Unemployed	7	29.2
Length of stay in care (years)		
1.5	4	16.7
**2**	**12**	**50.0**
3	8	33.3

## Data Availability

The data generated during or analyzed during this study are not publicly available in order to protect patient privacy. Data are available from Omoladun O. Odediran, email: ladun812@gmail.com, for researchers who meet criteria for access to confidential data.

## Supplementary Materials

Supplementary Materials The semistructured interview guide used to carry out the interviews with our study participants. Completed SRQR checklist.
