# Supplementary material for: A Qualitative Study Exploring Factors Associated with Retention in HIV Care among Women with HIV in a Large HIV Clinic in Lagos, Nigeria, after Implementing the Test and Treat Policy
Source: AIDS Res Treat. 2022 Aug 9;2022:9074844. doi: 10.1155/2022/9074844 (PMC9381265; doi:10.1155/2022/9074844)
Supplement: Supplementary Materials — The semistructured interview guide used to carry out the interviews with our study participants. Completed SRQR checklist. [file 9074844.f1.zip › INTERVIEW GUIDE.docx]

**INTERVIEW GUIDE**

Interviewer’s name:

Time started: Time ended:

**Statement of consent**

The interviewer has explained to me in a language that I understand, and I understand the process and the purpose of the interview the benefits of the study. I understand my right to voluntary participation and withdrawal at any stage of the study if I wish to do so.

**DATE: _________________**

**______________________________________________________________________________**

**Section 1: Demographics**

1. Age _______ years
2. Occupation: _______________
3. Employment Status: ____________________________________________
4. Marital Status: a) single b) Married c) Divorced e) Widowed

Other specify________

1. Religion: a) Christianity b) Islam c) Traditional d) Others (specify) _________________________
2. Ethnicity: a) Yoruba b) Igbo c) Hausa d) Others (specify) _________________________
3. Level of education: a) No formal education b) Primary school uncompleted c) Primary school completed d) Secondary school uncompleted e) Secondary school completed f) Post secondary (specify) _________
4. Length of stay in care__________

**Section 2: Knowledge Assessment**

1. Do you think that there are benefits for taking the HIV drugs for someone that is HIV positive?

- If yes, what are the perceived benefits to the patient herself? And to the people around them?
- Are there any disadvantages of taking HIV drugs? If yes, what are they? ( ask this question even if they mention benefits)

1. How often do you think a woman living with HIV should take her HIV medication?

**Self Assessment**

1. How did you feel when you were first diagnosed with HIV?
2. How soon after your diagnosis were you offered ART?
3. How has taking HIV medication being for you?

- Are there things you consider important to you before you accepted or rejected the HIV drugs?

**Section 3: Disclosure and Social support**

1. Does anyone in your household know about your HIV status?
2. Are there people that you rely on for social support?  Who are they?
3. How do you earn an income? Do you depend on spouse/partner or other family members and friends?
4. Are there people you trust enough to disclose your HIV status to? If yes, who?, and If No, Why?

**Section 4a: Ease of travelling to the clinic**

1. How easy is it for you to get to the clinic on appointment days?

- How long does it take to get to the clinic from your house?
- How much do you spend on transportation averagely on clinic days?

**Section 4b: Perception on care received at the clinic**.

1. In your opinion, will you say you have received adequate support from the HIV clinic? If yes, how? If no, how?
2. Are there any difficulties/ challenges you encounter in the process of accessing care at the clinic?

- If yes, what are these difficulties?
- How have you been able to overcome them, or manage with them?

1. In your opinion, what do you think can be improved on in the clinic

**Section 5:** **Intent to remain in care**

1. When you receive drugs at the clinic, what do you do first?

- If they start taking them- how soon do you start taking them after a clinic visit?
- How are you motivated to take the medication? (*probe- personal drive, focus on health, trust in the healthcare delivery personnel, support from family and friends*)

Thank you for your participation.
